# Adrenal Venous Sampling Aids in Distinguishing 17-Hydroxyprogesterone Hypersecreting Adrenal Cortical Adenomas from Non-Classical 21-Hydroxylase Deficiency

**DOI:** 10.3390/diagnostics16020202

**Published:** 2026-01-08

**Authors:** Ruojun Qiu, Tian Yang, Chengxin Shang, Weifen Zhu, Fenping Zheng

**Affiliations:** 1 Department of Endocrinology, The Affiliated Sir Run Run Shaw Hospital, College of Medicine, Zhejiang University, Hangzhou 310016, China; 3150103205@zju.edu.cn (R.Q.); shirley6613@163.com (C.S.); 3315014@zju.edu.cn (W.Z.); 2 Department of Endocrinology, The Fourth Affiliated Hospital, College of Medicine, Zhejiang University, Yiwu 322000, China; 8012023@zju.edu.cn

**Keywords:** 17-hydroxyprogesterone, adrenal cortical tumor, adrenal venous sampling, non-classic congenital adrenal hyperplasia

## Abstract

**Background and Clinical Significance:** This report presents the case of a 33-year-old female with recurrent miscarriage, investigated for an adrenal cortical adenoma characterized by autonomous secretion of 17-hydroxyprogesterone (17-OHP). The findings challenge the established diagnostic paradigm, which predominantly attributes elevated serum 17-OHP to congenital adrenal hyperplasia (CAH) or non-classical CAH (NCCAH). **Case Presentation:** The patient was found to have elevated serum 17-OHP and a 2 cm left adrenal mass. Normal testosterone and precursor levels, along with whole-exome sequencing (WES), argued against a diagnosis of non-classical 21-hydroxylase deficiency (NC-21OHD). An ACTH stimulation test elicited a mild-to-moderate rise in 17-OHP, while adrenal venous sampling (AVS) confirmed marked lateralization of 17-OHP hypersecretion to the left side. Postoperative normalization of 17-OHP levels further supported the diagnosis of a 17-OHP-secreting tumor. Histopathological analysis identified tumor regions with non-uniformly high expression of CYP17A1 and CYP21A2. Preliminary transcriptomic profiling revealed that differentially expressed genes (DEGs) were enriched in microRNA-related and PI3K-Akt signaling pathways. **Conclusions:** This paradigm-shifting case indicates that, in addition to 21OHD, a 17-OHP-hypersecreting adrenal adenoma should be considered in the differential diagnosis of elevated 17-OHP. The integration of multimodal diagnostic techniques, particularly AVS, is valuable for localizing hormonally active tumors. Preliminary mechanistic insights suggest a potential role for epigenetic dysregulation in the pathogenesis of this tumor type.

## 1. Introduction

17-hydroxyprogesterone (17-OHP) is a key intermediate in steroid hormone biosynthesis. 17-OHP is efficiently converted to 11-deoxycortisol by the enzyme 21-hydroxylase, maintaining serum concentrations typically below 2 ng/mL. Disruption of this pathway leads to the accumulation of 17-OHP [1]. 21-hydroxylase deficiency (21OHD), which results in congenital adrenal hyperplasia (CAH), represents the most common cause of pathologically elevated 17-OHP [2]. The resultant cortisol/aldosterone deficiency and androgen excess manifest clinically as virilization and electrolyte disturbances in classic CAH, while non-classic 21OHD (NC-21OHD), which is caused by partial 21-hydroxylase deficiency, presents moderately elevated progesterone and 17-OHP and mild hyperandrogenic features overlapping with polycystic ovary syndrome (PCOS), including hirsutism, oligomenorrhea, and subfertility [3].

Classic CAH is rare, with an estimated prevalence between 1:10,000 and 1:20,000 in most populations. Non-classic CAH, however, is more common, affecting approximately 1:200 to 1:2000 individuals [4,5]. Screening for 21OHD is recommended when a basal follicular-phase 17-OHP level exceeds 6 nmol/L (2 ng/mL) [6]. A 17-OHP level greater than 30 nmol/L (10 ng/mL) after adrenocorticotropic hormone (ACTH) stimulation strongly suggests 21OHD, although most patients with NC-21OHD exhibit stimulated levels between 30 and 300 nmol/L [7]. Thus, the ACTH stimulation test is valuable for differentiating NC-21OHD from PCOS and for assessing the severity of 21OHD [8]. Genetic testing for CYP21A2 mutations serves as an important confirmatory diagnostic tool for NC-21OHD [9].

Accumulation of 17-OHP can be converted into active androgens which affect follicular development and ovulation by suppressing the hypothalamic-pituitary-ovarian (HPO) axis thereby impairing follicular development and ovulation. Furthermore, its conversion to progesterone can lead to abnormal endometrial development and cervical mucus, hindering sperm penetration and embryo implantation [10,11]. While an elevated serum 17-OHP level is a significant indicator for 21OHD, its specificity is limited. Although chronic ACTH stimulation in CAH typically causes diffuse or nodular adrenal hyperplasia, the coexistence of an elevated 17-OHP level and an adrenal incidentaloma does not confirm 21OHD. In fact, while biochemical evidence suggestive of CAH has been reported in 5.9% of adrenal incidentaloma cohorts, only 0.8% were genetically confirmed as CAH [12]. Therefore, alternative diagnoses should be considered in patients with elevated 17-OHP, particularly those without clinical hyperandrogenism, who have normal androgen and precursor levels, and in whom CYP21A2 mutations are not identified.

Here, we present a rare case of a woman experiencing recurrent spontaneous miscarriages during early pregnancy. She was initially diagnosed with NC-21OHD but was eventually diagnosed with an adrenal cortical adenoma characterized by hypersecretion of 17-OHP. Adrenal venous sampling (AVS) played a crucial role in the diagnostic and therapeutic process. Additionally, we conducted preliminary explorations into the pathogenesis of this tumor.

## 2. Case Presentation

A 33-year-old woman presented with a history of two spontaneous miscarriages, both occurring at approximately 7 weeks of gestation in February and October of 2024. Notably, her menstrual cycles were regular (menarche at age 11), and she had no clinical signs of hyperandrogenism, such as hirsutism or acne. During preconception screening in September 2023, laboratory tests revealed subclinical hypothyroidism with positive thyroid peroxidase antibodies (TPOAb), for which levothyroxine supplementation was initiated; subsequent thyroid-stimulating hormone (TSH) levels remained <2.5 mIU/L. Following her first miscarriage in July 2024, further evaluation led to diagnoses of antiphospholipid syndrome (based on a positive ANA titer of 1:80 and elevated anticardiolipin antibodies), impaired glucose tolerance, and mild insulin resistance. Treatment with hydroxychloroquine and metformin was subsequently started. Further laboratory tests at that time showed an isolated elevation of 17-OHP to 9.18 ng/mL, while ACTH and cortisol levels followed a normal diurnal rhythm, and both progesterone and testosterone levels were within normal limits. This prompted initiation of empiric therapy with glucocorticoids (dexamethasone, later switched to prednisone) and the anti-androgen spironolactone. After treatment with prednisone at a dose of 5–2.5 mg twice daily, her 17-OHP level normalized to 0.8965 ng/mL.

**Figure 1:**
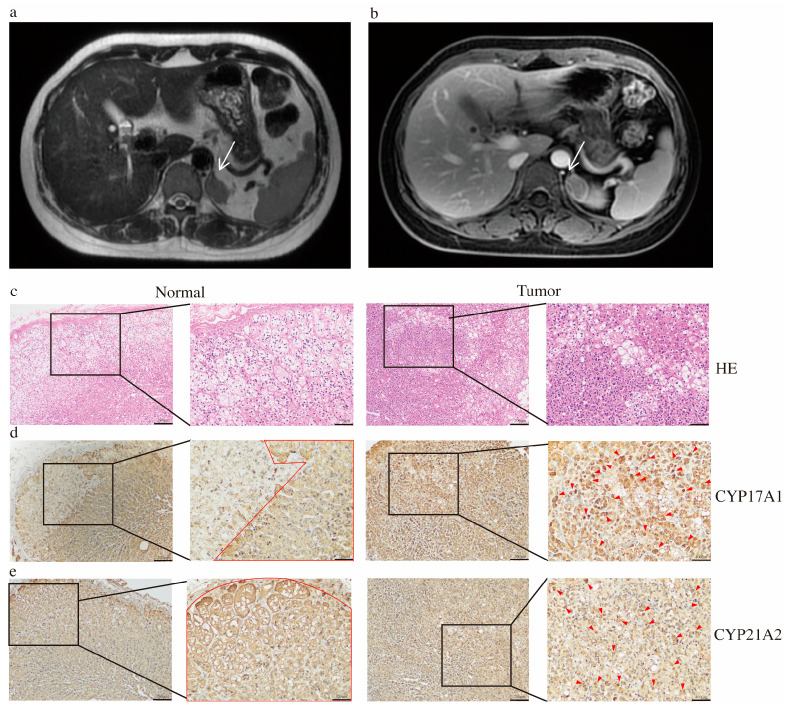
Imaging and pathological examination of the adrenal tumor: T2-weighted MRI (**a**) and contrast-enhanced MRI with AX delay (**b**) showing left adrenal gland mass measuring approximately 25 × 16 mm (arrow); (**c**) hematoxylin and eosin stain of peritumor adrenal tissue (“Normal”) and adrenal tumor (“Tumor”), scale bars: 100–200 μm; and immunohistochemical analyses of CYP17A1 (**d**) and CYP21A2 (**e**), scale bars: 100–200 μm. Red lines outline CYP17A1/CYP21A2-positive regions in normal adrenal glands and red triangular arrows indicate those in tumor tissues.

**Figure 2:**
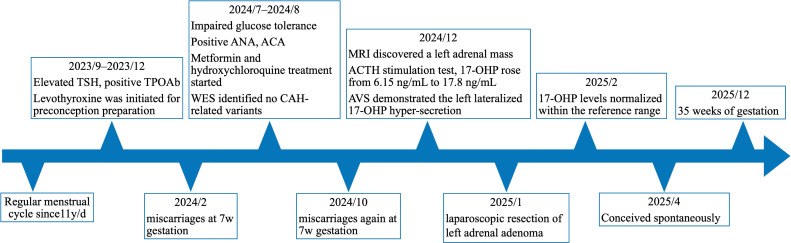
Timeline of the treatment course. TSH, Thyroid-Stimulating Hormone; TPOAb, Thyroid Peroxidase Antibody; ANA, Antinuclear Antibody; ACA, Anticardiolipin Antibody; WES, Whole-Exome Sequencing; CAH, Congenital Adrenal Hyperplasia; MRI, Magnetic Resonance Imaging; ACTH, Adrenocorticotropic Hormone; 17-OHP, 17-Hydroxyprogesterone; AVS, Adrenal Vein Sampling.

Despite taking prednisone, she unfortunately suffered a second spontaneous miscarriage at 7 weeks gestation. Then she was referred to our endocrine outpatient clinic in December 2024. Her paternal grandmother had a history of diabetes. Her father suffered from diabetes and hypertension, and died of cerebral infarction. Her mother had no history of miscarriages, and there was no family history of adrenal tumors. Both parents denied consanguineous marriage. Further laboratory results indicated elevated basal 17-OHP and slightly elevated progesterone, with normal levels of estrogen, androgen and its precursors, cortisol, aldosterone, and other relevant measurements (Table 1). Further in an ACTH stimulation test, 17-OHP rose from 6.15 ng/mL (baseline) to 17.8 ng/mL (Table 2). No variants linked to miscarriage or CAH were identified by WES of peripheral blood samples. Surprisingly, adrenal MRI uncovered a left adrenal mass (25 × 16 mm) (Figure 1a,b). To directly ascertain whether the adrenal mass exhibits high secretion of 17-OHP, we performed AVS, a technique typically employed as the gold standard for subtyping primary aldosteronism (PA). The AVS results demonstrated the left lateralized 17-OHP hypersecretion most-likely originating from the left adrenal mass, with a dominant side ratio of 32.4:1 (Table 3, detailed AVS procedures and interpretation were provided in the Supplementary Materials). On the second day following the laparoscopic resection of her left adrenal adenoma, her 17-OHP levels dropped to 0.35 ng/mL, and one-month post-surgery, it was 0.31 ng/mL, which directly supported the diagnosis of an adrenal adenoma with high 17-OHP secretion. The patient achieved spontaneous conception 3 months postoperatively and was at 35 weeks of gestation at the follow-up in December 2025 with an uneventful pregnancy course. The timeline of treatment course is shown in Figure 2.

The postoperative pathological examination of the specimen confirmed an adrenocortical adenoma (Figure 1c). Histological evaluation with H&E staining revealed well-defined tumor cell borders without evidence of capsular invasion. The tumor cells were slightly smaller than those of the adjacent adrenal cortex, with prominent nucleoli and an increased nuclear-to-cytoplasmic ratio. Some cells exhibited abundant, foamy cytoplasm suggestive of lipid accumulation. Focal nuclear pleomorphism was present, with scattered cells showing high-grade nuclear features.

Further immunohistochemistry revealed heterogeneous CYP17A1 and CYP21A2 expression in tumor tissue. In normal adrenal tissue, CYP17A1 staining is predominantly expressed in the fasciculata and reticularis zones of the adrenal cortex. However, the expression of CYP17A1 within tumors is heterogeneous, with strong expression within certain areas (Figure 1d). For CYP21A2, normal adrenal tissue shows moderate cytoplasmic staining, primarily localized to the fasciculata and glomerulosa zones of the adrenal cortex. In adrenal tumors, some tumor cell clusters show strengthened positive staining, while other areas are weak staining (Figure 1e).

**Figure 3:**
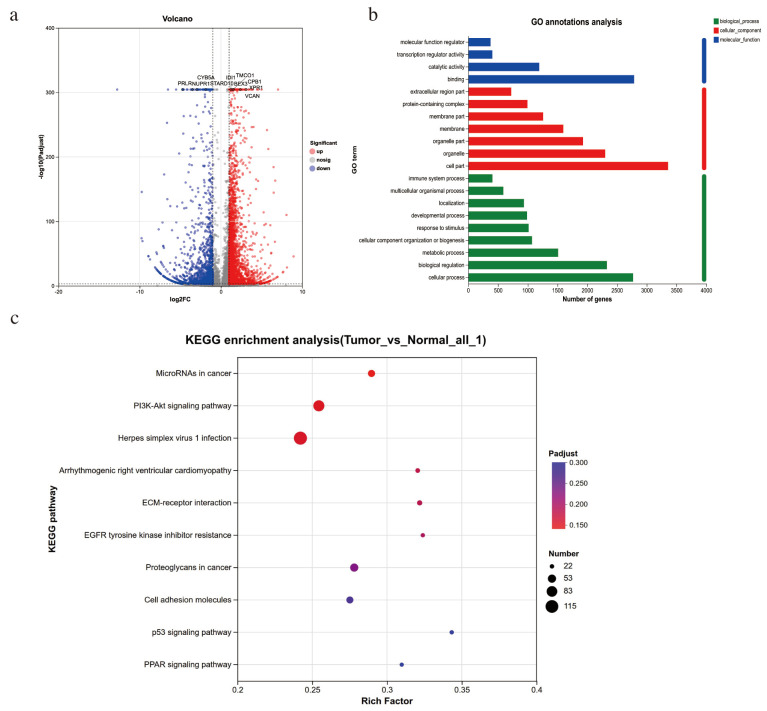
Differences in transcriptomics between peritumor adrenal tissue (“Normal”) and adrenal tumor (“Tumor”): (**a**) Volcano plot of DEGs; (**b**) GO annotation analyses; and (**c**) KEGG enrichment analyses.

To explore the preliminary molecular mechanisms of adrenal cortical adenoma with high secretion of 17-OHP, we performed transcriptomic sequencing on tumor tissue (“Tumor”) and adjacent relative normal adrenal tissues (“Normal”). Bioinformatics analysis of the transcriptomic data identified 4208 differentially expressed genes (DEGs) using |log2 foldchange| ≥ 1 and P_adjust_ < 0.001. As shown in the Volcano plot (Figure 3a), there were 2813 upregulated and 1395 downregulated genes. GO analysis showed that the biological process terms were mainly the metabolic process, cellular process, and biological regulation (Figure 3b). KEGG enrichment analysis indicated that these DEGs were primarily enriched in pathways related to “MicroRNAs in cancer”, “PI3K-Akt signaling pathway”, and “ECM-receptor interaction”, all of which are closely associated with tumor development (Figure 3c). Further screening and analysis of differential genes identified two clusters of the most significantly differentially expressed genes: cytochrome b5 type A (CYB5A) and steroidogenic acute regulatory protein (STARD1), which are involved in steroidogenesis [13,14], and actin-related protein 2 (ACTR2) and insulin-like growth factor 2 (IGF2), which are associated with tumorigenesis and progression [15,16].

## 3. Discussion

We present a diagnostically challenging case of a 33-year-old woman with recurrent miscarriages and isolated 17-OHP elevation secondary to an autonomous 17-OHP-secreting adrenal adenoma. Key findings include the following: (1) ACTH-stimulated 17-OHP elevation (basal: 6.15 ng/mL; post-stimulation: 17.8 ng/mL) without CAH/NCCAH relevant gene mutations; (2) AVS-confirmed lateralized 17-OHP hypersecretion from a left adrenal mass; (3) rapid normalization of 17-OHP following tumor resection, along with uneventful spontaneous conception postoperatively; and (4) local over-expression of CYP17A1 in tumor tissues may be responsible for the isolated 17-OHP elevation and the oncogenesis potentially mediated by epigenetic mechanisms. This case demonstrates the diagnostic dilemma caused by adrenal tumors that mimic the steroidogenic profile of NC-21OHD, and further outlines the two primary clinical significances of this study: (1) it suggests in women with menstrual irregularities and/or a history of abnormal pregnancies, besides PCOS and CAH, rare conditions such as 17-OHP-secreting adrenal incidentalomas should also be considered, particularly when the steroid hormone profile and genetic testing results are inconsistent with the former two diagnoses; (2) it demonstrates that a multimodal diagnostic approach, especially the use of AVS, can be effectively extended beyond its established role in subtyping PA to facilitate the diagnosis of rare steroid-secreting adrenal tumors.

Persistent elevation of 17-OHP in women with infertility or pregnancy loss necessitates a comprehensive diagnostic evaluation [17]. While 21-OHD remains the leading cause of pathologic 17-OHP elevation, the patient’s biochemical profile shows some inconsistencies: (1) Absence of hyperandrogenism. The patient lacks clinical signs (e.g., menstrual irregularity, hirsutism, or acne) or biochemical evidence of elevated circulating androgens. (2) Normal steroid precursor levels. Both 17-OHP precursors (progesterone) and androgen precursors (DHEAS and androstenedione) remain normal. (3) Negative genetic evidence. WES failed to identify pathogenic variants associated with CAH/NCCAH from the peripheral blood samples [18]. (4) Atypical ACTH stimulation response. Previous studies have indicated that patients with NC-21OHD typically exhibit a median basal 17-OHP level of 6–10 ng/mL, with post-ACTH stimulation levels usually exceeding 30 ng/mL [19,20,21]. While our case demonstrated a post-ACTH 17-OHP level of 17.8 ng/mL, a relatively weaker response compared to NC-21OHD profiles, indicating a moderate autonomy in the secretion of 17-OHP of this tumor. The patient was complicated with other comorbidities potentially associated with miscarriage, such as positive TPOAb and anticardiolipin antibodies, impaired glucose tolerance, and insulin resistance. However, the impact of these conditions on miscarriage was significantly lower than that of the elevated 17-OHP, which exhibits a progesterone-like effect. On the other hand, despite corresponding treatments of these comorbidities, miscarriage could not be prevented. The successful pregnancy achieved after addressing the elevated 17-OHP further supports its stronger correlation with the patient’s history of miscarriage [22,23].

AVS, while predominantly employed for lateralization of aldosterone hypersecretion in PA, has also been utilized in attempts to identify the dominant side with high cortisol secretion, including primary pigmented nodular adrenocortical disease (PPNAD), ACTH-independent macronodular adrenal hyperplasia (AIMAH), and bilateral adrenal adenomas [24,25,26,27]. For the first time, we report the novel application of AVS for determining lateralized 17-OHP hypersecretion in a diagnostically challenging case. In 21-OHD, AVS would theoretically demonstrate bilateral adrenal 17-OHP concentrations exceeding twice peripheral levels (adrenal vein/inferior vena cava (IVC) ratio ≥ 2:1) with inter-adrenal symmetry (e.g., the bilateral adrenal veins ratio ≤ 4:1) [28,29]. Our patient exhibited striking lateralization with left adrenal 17-OHP levels at 32.4-fold higher than the right adrenal vein, supporting the exclusion of 21-OHD. AVS confirmed the significantly elevated 17-OHP levels localized to the left adrenal mass, with a rapid post-resection decline directly supporting autonomous 17-OHP secretion by the tumor in this case. Notably, while previously reported cases of adrenal cortical adenomas with autonomous 17-OHP secretion have typically shown resistance to exogenous steroid suppression [30], our patient demonstrated suppressible 17-OHP levels in response to low-dose prednisone. This may be related to the fact that some adrenal adenomas still retain a certain degree of responsiveness to ACTH, which is consistent with the observation in our case where 17-OHP showed a certain degree of increase following ACTH stimulation.

At present, AVS remains the most direct and reliable method for localizing tumors and assessing their secretory activity, as it allows direct measurement of hormone levels from the adrenal veins. This technique is well-established, for instance, in lateralizing aldosterone secretion in PA. Although multiparametric imaging approaches, including radionuclide-labeled techniques (e.g., CXCR4 and MIBG), have been widely adopted in clinical practice for adrenal tumor detection [31,32], no effective radionuclide-labeled imaging method has been reported for 17-OHP-secreting incidentalomas. In contrast, AVS provides a direct and effective approach for determining the lateralization of hormone hypersecretion from such tumors.

In a previously reported case of 17-OHP secretion tumor, diagnosis was established via routine hormonal assessments combined with CT/MRI scans [30,33]. Our case broadens the pathological spectrum of adrenal tumors and expands the clinical utility of AVS to the specific steroidogenic tumors’ detection. We further performed tumor transcriptomic profiling, which provide preliminary insights into the patho-physiological mechanisms. In our patient, histopathological analysis revealed focal over-expression of CYP17A1 within the tumor, accompanied by mild compensatory up-regulation of CYP21A2. This enzymatic imbalance likely drives the elevated 17-OHP levels in the left adrenal vein/peripheral, as well as the intra-adrenal gradient, consistent with autonomous 17-OHP hypersecretion rather than 21-OHD. However, the underlying mechanism by which elevated 17-OHP fails to upregulate androgens and their precursors remains unclear, and is presumed to be linked to the structural incompleteness of tumor-autonomously secreted 17-OHP. The patient had no family history of adrenal tumors, and no variant genes linked to CAH/NCCAH were detected by peripheral blood WES. Transcriptomic analysis suggests epigenetic modifications as a potential driver of the tumorigenesis.

However, this study also has certain limitations. First, somatic mutation testing was not performed on the tumor, rendering the molecular conclusions largely speculative. Second, the single-case study design inherently limits the generalizability of our findings and precludes causal inference. Third, functional steroidogenic assays were not compressively performed.

## 4. Conclusions

Adrenal cortical adenomas with autonomous 17-OHP secretion do exist and should be considered as a differential diagnosis in individuals with elevated 17-OHP, particularly among women with infertility or recurrent miscarriage. AVS offers considerable value in identifying and differentiating rare hormone-secreting tumors. Epigenetic dysregulation might contribute to the pathogenesis of autonomous 17-OHP-secreting adenomas, though more clinical data and fundamental research are required for validation.

## Figures and Tables

**Table 1:** Baseline laboratory test and oral glucose tolerance test.

		Measured Value		Reference Range	
17-OHP (ng/mL)		9.41		0–1.85 (follicular phase)	
Estradiol (pg/mL)		71.73		29.42–442.62	
LH (IU/L)		19.9		19.18–103.03	
FSH (IU/L)		4.99		4.54–22.51	
Progesterone (μg/L)		1.64		0.31–1.52 (follicular phase)	
Prolactin (μg/L)		16.73		3.34–26.72	
Testosterone (μg/L)		0.52		0.1–0.75	
DHEA-S (μg/dL)		144		23–266	
Dihydrotestosterone (pg/mL)		267		<300	
Androstenedione (ng/mL)		1.03		0.3–2	
Aldosterone (ng/dL)		18.7		3.0–35.3	
Direct Renin Concentration (μIU/mL)		32.28		4.4–46.1	
Cortisol (8 a.m.) (μg/dL)		17.24		6.7–22.6	
Cortisol (4 p.m.) (μg/dL)		5.03		0–10	
ACTH (8 a.m.) (ng/L)		24		10–80	
ACTH (4 p.m.) (ng/L)		5		5–40	
24 hr UFC (calculated) (μg/24 h)		135.8		58.0–403.0	
Potassium (mmol/L)		3.92		3.5–5.3	
Sodium (mmol/L)		141		137–147	
HbA1c %		5.2		4.0–6.0	
OGTT	0 h	0.5 h	1 h	2 h	3 h
Glucose (mmol/L)	5.87	10.03	11.36	9.69	6.03
Insulin (mIU/mL)	14.3	46.7	76.7	124.9	84.6
C-peptide (ng/mL)	2.82	6.22	10.08	14.73	12.06

17-OHP, 17-hydroxyprogesterone; LH, luteinizing hormone; FSH, follicle-stimulating hormone; DHEA-S, dehydroepiandrosterone sulfate; ACTH, adrenocorticotropic hormone; UFC, urinary free cortisol; HbA1c, Glycated Hemoglobin; OGTT, oral glucose tolerance test. Based on the patient’s menstrual cycle and last menstrual period, the patient was in the follicular phase when the blood sample was taken.

**Table 2:** ACTH-stimulated test (8 h continuous intravenous drip administration).

	Before Stimulate	After Stimulate
ACTH (ng/L)	22	>2000
Cortisol (ug/dL)	14.84	35.95
24 hr UFC (calculated) (μg/24 h)	135.8	>90,000
17-OHP (ng/mL)	6.15	17.8
DHEA-S (ng/mL)	1380	1880
Dihydrotestosterone (pg/mL)	181	278
Androstenedione (ng/mL)	0.7	1.46
Testosterone (μg/L)	0.4	0.58

UFC, urinary free cortisol; 17-OHP, 17-hydroxyprogesterone; DHEA-S, dehydroepiandrosterone sulfate. 8 h continuous intravenous drip administration: 25 IU of corticotropin in 500 mL of 5% glucose solution for continuous intravenous infusion over 8 h. Blood samples was collected before and after the infusion for testing. 17-OHP, DHEAs, androstenedione, and dihydrotestosterone are measured using mass spectrometry (LC-MS/MS), while other steroid hormones detection was performed via chemiluminescence immunoassay on the Access Immunoassay System (DXI800, Beckman Coulter, Brea, CA, USA), employing the reagent kits supplied by the manufacturer.

**Table 3:** Adrenal venous sampling.

	IVC	LAV	RAV	LAV/IVC	RAV/IVC	LAV/RAV
17-OHP (ng/mL)	11.5	798	23.1	69.5	2.0	34.5
DHEA-S (ng/mL)	566	954	2460	-	-	-
Dihydrotestosterone (pg/mL)	96.4	157	216	-	-	-
Androstenedione (ng/mL)	1.48	40.3	42.9	-	-	-
Cortisol (μg/dL)	20.74	385.81	361.83	18.60	17.45	1.1
17-OHP/Cortisol	0.5545	2.0684	0.0638	-	-	32.4

IVC, inferior vena cava; LAV, left adrenal vein; RAV, right adrenal vein; 17-OHP, 17-hydroxyprogesterone; DHEA-S, dehydroepiandrosterone sulfate. Cortisol concentrations were corrected for a 1:10 dilution factor (multiplied by 10).

## Data Availability

The original contributions presented in the study are included in the article/Supplementary Materials. The datasets generated during the current study are available in the Genome Sequence Archive (GSA) database repository, with the accession number HRA011736 and are available at the following URL: https://ngdc.cncb.ac.cn/gsa-human/ (accessed on 28 October 2025).

## Supplementary Materials

The following supporting information can be downloaded at: https://www.mdpi.com/article/10.3390/diagnostics16020202/s1, Description of the materials and methodology involved in this study.
