# Supplementary material for: Adrenal Venous Sampling Aids in Distinguishing 17-Hydroxyprogesterone Hypersecreting Adrenal Cortical Adenomas from Non-Classical 21-Hydroxylase Deficiency
Source: Diagnostics (Basel). 2026 Jan 8;16(2):202. doi: 10.3390/diagnostics16020202 (PMC12840385; doi:10.3390/diagnostics16020202)
Supplement: Supplementary file 1 [file diagnostics-16-00202-s001.zip › diagnostics-3985446-supplementary.pdf]

Supplement Materials & Methods

1. Whole - exome sequencing (WES):

In brief, after clinical indication assessment and informed consent acquisition, peripheral blood was collected, followed by genomic DNA extraction and quality control. Then, library construction is completed through DNA fragmentation, adapter ligation and exon probe capture, and the qualified libraries after PCR amplification and quality control are subjected to high - throughput sequencing on the platform. Subsequently, raw data are filtered and aligned, and candidate variants are screened by annotation with relevant databases. Finally, variant sites are verified by Sanger sequencing, pathogenicity classification is conducted in accordance with ACMG/AMP criteria.

2. AVS:

In brief, following routine disinfection, sterile draping, and local anesthesia with 2% lidocaine, bilateral femoral veins were punctured using an 18-gauge needle via the modified Seldinger technique (a minimally invasive vascular access method involving sheath advancement over the needle prior to guidewire and device insertion). A 5-Fr sheath was placed bilaterally. Under fluoroscopic guidance, a 5-Fr Sim-2 catheter and a 5-Fr C2 catheter were selectively advanced into the right and left adrenal veins, respectively. Venography confirmed the right adrenal central vein draining directly into the inferior vena cava with the characteristic octopus-like morphology, whereas the left adrenal vein emptied into the left renal vein. For the critical step of bilateral simultaneous sampling, paired microcatheters were used to draw 5-mL blood aliquots from both adrenal veins at the exact same time point. Matching 5-mL samples were also collected from the bilateral renal veins and inferior vena cava for comparative analysis. After sample collection, catheters and sheaths were removed. Hemostasis was achieved via manual compression at the puncture sites, with no bleeding or hematoma observed, followed by pressure dressing application. Successful catheterization during AVS was confirmed by a cortisol gradient (adrenal vein [AV] to peripheral vein [PV]) exceeding 2:1 bilaterally. For the assessment of 17-OHP secretion, interpretation was based on the absolute 17-OHP concentrations in bilateral adrenal and peripheral veins, as well as the 17-OHP to cortisol (17-OHP/Cor) ratio on each side. Lateralization was considered present when the ratio of these 17-OHP/Cor ratios between the two sides exceeded 4, applying a lateralization index (LI) analogous to that used in AVS for primary aldosteronism.

3. Histologic analyses:

Tumor tissues and adjacent relatively normal adrenal tissues were fixed in 4% paraformaldehyde, dehydrated through an ethanol gradient, and embedded in paraffin blocks. Serial sections of 4 μm thickness were cut using a microtome, mounted on glass slides, and stained with hematoxylin and eosin (H&E) for morphological analysis. Histopathological images were acquired using an optical microscope (Nikon).

4. Immunohistochemical analyses:

Tissue sections were incubated with secondary antibodies after being stained with CYP17A1 (PA006392EA01HU, Cusabio, Wuhan China, diluted at 1:100) and CYP21A2 (PA006400LA01HU, Cusabio, Wuhan China, diluted at 1:100) primary antibodies at 4°C overnight. After that, sections were stained with diaminobenzidine to produce images of the tissues under a fluorescence microscope (Nikon).

5. Transcriptomics analysis:

Total RNA was extracted from the tissue using TRIzol® Reagent according the manufacturer's instructions. Then RNA quality was determined by 5300 Bioanalyser (Agilent) and quantified using the ND-2000 (NanoDrop Technologies). RNA purification, reverse transcription, library construction and sequencing were performed at Shanghai Majorbio Bio-pharm Biotechnology

Co., Ltd. (Shanghai, China) according to the manufacturer's instructions. The RNA-seq transcriptome library was prepared following Illumina® Prep, Ligation (San Diego, CA) using 1μg of total RNA. To identify DEGs (differential expression genes) between two different samples, the expression level of each transcript was calculated according to the transcripts per million reads (TPM) method. RSEM was used to quantify gene abundances. Essentially, differential expression analysis was performed using the DEGseq. DEGs with |log2 foldchange|≧1 and $P_{adjust} < 0.001$.
